# Pyruvate dehydrogenase kinase regulates macrophage polarization in metabolic and inflammatory diseases

**DOI:** 10.3389/fimmu.2023.1296687

**Published:** 2023-12-18

**Authors:** Chenyu Li, Chuanbin Liu, Junfeng Zhang, Yanyu Lu, Bingtong Jiang, Huabao Xiong, Chunxia Li

**Affiliations:** ^1^ Institute of Immunology and Molecular Medicine, Jining Medical University, Jining, Shandong, China; ^2^ Department of Pediatric Dentistry, Jining Stomatological Hospital, Jining, Shandong, China

**Keywords:** pyruvate dehydrogenase kinase, macrophage, polarization, metabolic reprogramming, inflammation

## Abstract

Macrophages are highly heterogeneous and plastic, and have two main polarized phenotypes that are determined by their microenvironment, namely pro- and anti-inflammatory macrophages. Activation of pro-inflammatory macrophages is closely associated with metabolic reprogramming, especially that of aerobic glycolysis. Mitochondrial pyruvate dehydrogenase kinase (PDK) negatively regulates pyruvate dehydrogenase complex activity through reversible phosphorylation and further links glycolysis to the tricarboxylic acid cycle and ATP production. PDK is commonly associated with the metabolism and polarization of macrophages in metabolic and inflammatory diseases. This review examines the relationship between PDK and macrophage metabolism and discusses the mechanisms by which PDK regulates macrophage polarization, migration, and inflammatory cytokine secretion in metabolic and inflammatory diseases. Elucidating the relationships between the metabolism and polarization of macrophages under physiological and pathological conditions, as well as the regulatory pathways involved, may provide valuable insights into the etiology and treatment of macrophage-mediated inflammatory diseases.

## 1 Introduction

Macrophages, the main effector cells of the innate immune response, are highly plastic and polarize into two phenotypes based on their microenvironment, namely classically activated (M1) and alternatively activated (M2) macrophages (1–3). The M1 phenotype primarily exerts inflammatory responses by secreting inflammatory factors, such as tumor necrosis factor (TNF)-α, interleukin (IL)-1β, IL-6, nitric oxide (NO), and reactive oxygen intermediates, to remove antigenic foreign substances from the host (4). The M2 phenotype performs inflammatory clearance and tissue repair by secreting IL-10, IL-13, transforming growth factor-β, and other anti-inflammatory molecules (5). Metabolites, oxygen tension, cytokines, inflammatory signals, and other factors of the tissue microenvironment play definitive roles in macrophage metabolic reprogramming, phenotype, and immune function. Moreover, the metabolic state of macrophages may directly or indirectly influence their phenotype and function (6, 7).

## 2 PDK

**Figure 1:**
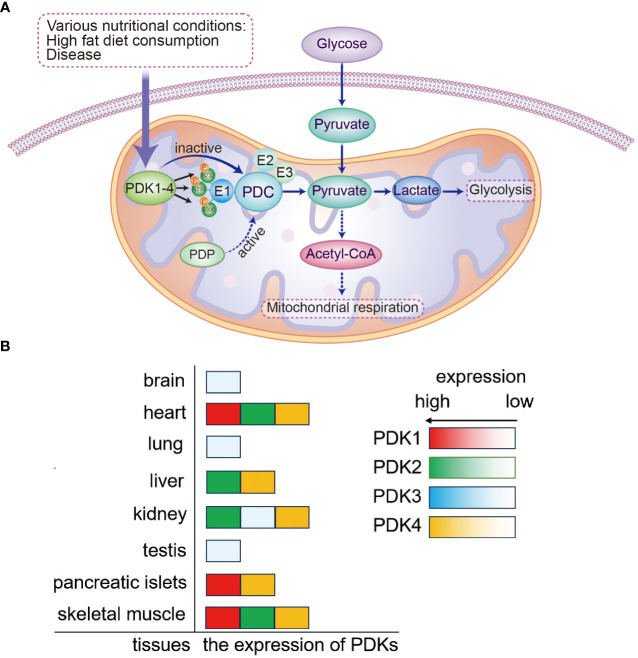
Mechanism of PDK-mediated regulation of PDC and the expression of PDKs. **(A)** Mitochondrial PDKs negatively regulate PDC activity by reversible phosphorylation and further link glycolysis to the tricarboxylic acid cycle. PDC activity is governed by reversible phosphorylation and dephosphorylation of its E1α subunit. The process of PDC inactivation is mediated by PDK phosphorylation of three E1α serine residues (Ser293, Ser300, and Ser232). **(B)** The expression of PDKs is shown in various tissues. PDK, pyruvate dehydrogenase kinase; PDP, pyruvate dehydrogenase phosphatase; PDC, pyruvate dehydrogenase complex.

The pyruvate dehydrogenase complex (PDC) is one of the main regulators of mammalian metabolic activity; in mitochondria, it consists of pyruvate dehydrogenase (E1), dihydrolipoic acid acetyltransferase (E2), dihydrolipoic acid dehydrogenase (E3), and E3-binding protein (E3BP) (8, 9). The PDC participates in the aerobic oxidation of glucose by catalyzing the oxidative decarboxylation of pyruvate to generate acetyl-CoA and NADH. PDC activity is primarily modulated by the four isozymes of PDK (PDK1–4) (10, 11). PDK1–4 inhibit PDC activity and downstream energy metabolism by phosphorylating specific serine residues (Ser293, Ser300, and Ser232) of the PDC E1α subunit; the PDC E1α subunit can be activated by dephosphorylation, which is catalyzed by pyruvate dehydrogenase phosphatase (10, 11). PDK1–4 sequences are up to 70% homologous and show differences only in their tissue distribution. Although PDK1 is abundant in the heart, skeletal muscle, and pancreatic islets, its expression levels are low in other tissues. PDK2 is relatively widespread and is highly expressed in the heart, kidney, liver, and skeletal muscle but shows lower expression in other tissues. PDK3 is only weakly expressed in some tissues, including the lung, kidney, testis, and brain; PDK4 is highly expressed in the heart, skeletal muscle, liver, kidney, and pancreatic islets (8, 12, 13). Thus, it is established that these four enzymes are distributed differently in various tissues (**Figure 1**).

Pyruvate decarboxylation is a critical step in cellular metabolism. Under normal physiological conditions, pyruvate dehydrogenase phosphatase activates the PDC, which subsequently catalyzes pyruvate in the tricarboxylic acid cycle to generate large quantities of ATP to satisfy the energy demands of the body. However, when the body is in a starved state, the PDC is deactivated by PDK phosphorylation, resulting in pyruvate decarboxylation disorders and accumulation of pyruvate in the cytoplasm. During starvation, pyruvate can be used as a precursor for glucose synthesis via the gluconeogenic pathway and can participate in the maintenance of blood glucose levels. Compared with that in normal tissues, the expression of PDK isoforms can be differentially upregulated in different tumor tissues under the induction of various factors, and the inhibition of PDC activity causes enhanced glycolysis in tumor cells (14–17). Under certain pathological conditions, the PDC undergoes phosphorylation, followed by inactivation, due to the action of PDK, and pyruvate cannot be completely oxidized or translated into fatty acids. In contrast, the malignant transformation of cells or alteration of metabolic pathways can continually activate PDK (18, 19) and cause disorders affecting glucose energy metabolism; this leads to tumors, diabetes, heart disease, and other related diseases. Thus, regulating the metabolic state of cells by inhibiting PDK activity can be an attractive strategy for the treatment of tumors, diabetes, heart disease, and other related diseases.

Pyruvate dehydrogenase kinase (PDK) is an important enzyme that regulates glucose metabolism. Recent research has shown that PDK controls the modulation of macrophage polarization and affects the pro-inflammatory and anti-inflammatory functions of macrophages. PDK inhibitors can enhance anti-inflammatory responses and effectively improve various chronic inflammatory diseases (20). Herein, we review the regulation of macrophage phenotypes and polarization by PDK and the potential underlying molecular mechanisms.

## 3 Macrophage polarization

Macrophages are innate immune cells with strong phagocytic and antigen-presenting functions; they are the most well-known members of the innate immune system and play a critical role in inflammation, tissue repair, tumor surveillance, and systemic metabolism (21–23). Macrophages are differentiated monocytes and are highly plastic. When unpolarized, in the resting state, they are identified as the inactive (M0) phenotype. Upon activation by different stimuli, macrophages can be transformed into two active phenotypes, namely classically activated (M1) and alternatively activated (M2). When stimulated by exogenous lipopolysaccharide (LPS), cytokine interferon (IFN)-γ, viruses, and exogenous DNA, macrophages tend to transition toward the pro-inflammatory phenotype and polarize into the M1 type, exerting pro-inflammatory and microbicidal effects by producing massive amounts of TNF-α, IL-1, NO, and other pro-inflammatory factors (1, 4, 24). Macrophages can be polarized to the M2 type after activation by IL-13 or IL-4, which are produced by immune cells (25, 26). Recent evidence suggests that M2 macrophages can be further subdivided into more subtypes (M2a–d), owing to differences in their activation conditions (27–29). The overall cellular functions of M2 macrophages involve regression of inflammation and wound healing. Thus, macrophage polarization directly affects the pathogenesis of related diseases.

Factors in the local tissue microenvironment, such as cytokines, inflammatory signals, and metabolites, determine the metabolic state of macrophages and their functional plasticity (30). Macrophage plasticity and immune function are closely related to alterations in their metabolism. For example, the “Warburg effect” refers to the theory that tumor cells undergo substantial metabolic reprogramming, consume large amounts of glucose, and produce lactate, even if glycolysis is still ongoing at sufficient oxygen levels (31, 32). A metabolic state similar to that of the “Warburg effect” also exists in activated M1 macrophages, which manifests as a reduction in oxidative phosphorylation (OXPHOS) and ATP synthesis and increased glucose consumption and lactate production. This state is accompanied by high expression of key enzymes involved in glycolysis, such as hexokinase and glucose-6-phosphate dehydrogenase, and high production of reactive oxygen species (33, 34). Accumulating evidence suggests that activated M1 macrophages can provide the energy required by cells for rapid pathogen eradication through the aerobic glycolytic pathway. However, activated M2 macrophages lack the metabolic phenotype of aerobic glycolysis; in M2 macrophages, pyruvate directly enters the tricarboxylic acid cycle and undergoes OXPHOS (30, 35).

Clearly, the switch in glycolytic metabolism can directly affect macrophage polarization and, in turn, their immune functions. Thus, interventions in macrophage metabolism are a promising direction for the amelioration of various inflammatory diseases by modulation of macrophage polarization.

## 4 Modulation of monocytes by PDK

As precursors for macrophages and dendritic cells, monocytes are differentiated from hematopoietic stem cells and develop in the bone marrow. After monocytes exude from blood vessels and migrate to different tissues and organs, they differentiate into macrophages. The PDC/PDK axis controls hypo-responsiveness in monocytes by regulating anabolic and catabolic energetics. In monocytes of septic mice and humans, high expression of PDK1 inactivates the PDC, leading to the formation of hypo-responsiveness in monocytes (36). In septic mice, PDK inhibition with dichloroacetic acid (DCA) leads to subsequent PDC activation, which enhances the mouse immune response, accelerates bacterial clearance, and improves survival. In addition, the production of mitochondrial oxidative bioenergy in isolated hepatocytes and splenocytes also increases (36). Unbiased metabolomics was applied to a severe acute inflammatory monocyte culture model simulating sepsis reprogramming, which showed that DCA reversed the inhibition of PDC activity. Consequently, inflammatory monocytes restored energy anabolism, tricarboxylic acid (TCA) cycle intermediates increased, and itaconate levels decreased (36, 37). However, only primary monocytes were examined in the study, and these new concepts have not been extended to studies on animal models or human inflammation-linked diseases, such as endotoxic shock. Further research is needed to prove that PDC/PDK linked to itaconate-induced tolerance and energy transformation is applicable to macrophages, dendritic cells, adaptive immune cells, or failing organs in humans.

Another study showed that inhibiting PDK expression using mitochondrial SIRT1 enhanced mitochondrial energy metabolism and terminated immune tolerance in monocytes via a previously unknown feedback mechanism (38). These findings indicate that PDK plays a crucial and currently unproven function in the regulation of mitochondrial bioenergetics in inflammatory stress, which may be improved to a certain extent by regulating PDK.

## 5 Regulation of macrophage orientations by PDK

### 5.1 Regulatory role of PDK1 in macrophage activation

PDK1 restricts the conversion of pyruvate to acetyl-CoA, which enters the TCA cycle and produces NADH, and blocks OXPHOS in mitochondria (39, 40). Recent findings provide substantial evidence of the function of PDK1 in macrophages. For example, Zheng et al. found that PDK1 promotes M1 macrophage polarization and suppresses M2 macrophage activation (41). After PDK1 deletion, LPS-induced aerobic glycolysis was attenuated in macrophages, and the number of M1 macrophages decreased. In addition, the production of M1 macrophage-associated inflammatory mediators IL-6, IL-12, IL-1β, and iNOS was markedly reduced. In comparison, it was observed that the activation of M2 macrophages was enhanced and accompanied by increased mitochondrial respiration during early activation. Furthermore, the expression levels of the M2 macrophage markers Arg1, YM-1, FIZZ-1, and MRC1 increased in response to IL-4-induced activation of macrophages after PDK1 knockdown. However, the level of STAT6 phosphorylation in macrophages did not change. Evidence suggests that PDK1 deletion attenuates glucose oxidation and does not affect the STAT6-mediated intracellular signaling pathway.

Macrophage activation and migration are two key functions contributing to the inflammatory process (42, 43). Recent studies have suggested potential correlations between hypoxia-induced metabolic switch and macrophage migration capacity. In hypoxia, PDK1 expression is increased in a hypoxia inducible factor (HIF)-α-dependent manner (44, 45). When the macrophage-derived cell line, RAW264.7, is subjected to mild hypoxia, the HIF-1α-PDK1 axis induces glycolytic reprogramming by converting pyruvate to lactate. As the HIF-1α-PDK1 axis is blocked by DCA, a chemical inhibitor of PDK, macrophage migration is markedly inhibited under hypoxic conditions; this may be attributed to the association of glycolysis with cytoskeletal actin remodeling. Cells migrate by rapidly depleting ATP in the cytoplasm to remodel cytoskeletal actin filaments (46). The infiltration of inflammatory immune cells, such as macrophages, into the inflamed microenvironment plays an important role in the pathogenesis of sepsis, atherosclerosis, and arthritis. Thus, influencing macrophage migration by regulating macrophage metabolism may be a novel therapeutic strategy for ameliorating inflammatory diseases.

### 5.2 Regulation of macrophage activation by PDK2

As an important regulator of glucose metabolism, PDK2 is associated with many diseases, such as tumor metabolic reprogramming, hepatic steatosis, and type 2 diabetes, and is closely correlated with immune cell dysregulation and inflammatory responses (47–49). The involvement of PDK2 in hepatic steatosis and cancer, via the regulation of glucose metabolism pathways, has been reported, and the upregulation of PDK2 expression in tumor cells is usually considered an important driver of metabolic rewiring (15, 50).

Macrophages play different roles in pathogen-induced immune responses, tissue repair, and maintenance of homeostasis. Thus, regulating PDK2 to alter macrophage metabolism and influence their activation or polarization has considerable therapeutic potential in cancer and inflammatory diseases (37, 51–53). The B7 family co-stimulatory molecule V-set and immunoglobulin domain-containing 4 (VSIG4) are complementary receptors of the immunoglobulin superfamily that are macrophage-specific surface molecules. VSIG4 mediates pathogen clearance by binding C3b or iC3b of the degradation component of complement C3, and previous studies have found that VSIG4 inhibits macrophage activation and M1 polarization by activating the PI3K/Akt-STAT3 pathway and upregulates PDK2 expression. Meanwhile, the downregulation of PDK2 expression enhances macrophage activation (54). Relevant experimental evidence shows that in PDK2 knocked down RAW264.7 cells or PDK2^-/–^BMDMs, LPS-induced mitochondrial oxidation and IL-6 and TNF-α levels are increased with high expression of CD40. Simultaneously, RAW264.7 cells with lentiviral overexpression of PDK2 exhibited the opposite effects. Moreover, pharmacological inhibition of PDK2 *in vivo* has been reported to markedly decrease the mortality of LPS-treated mice, alleviate liver and lung tissue damage, and downregulate pro-inflammatory factor expression. PDK2 regulates macrophage activation and pro-inflammatory factor production *in vitro* via TLR4-MAPK signaling cascades, which affects the occurrence and development of endotoxic shock (55). The above studies confirmed that PDK2 regulates macrophage polarization via different mechanisms by knocking down the PDK2 gene or modulating PDK2 expression using a lentivirus, which in turn affects the development of inflammation. The experimental protocols and reagents used in these studies may differ, which might explain their differing conclusions. It is worth noting that it may be more beneficial to research the role of PDK2 in activating the TLR4-MAPK signaling pathway in macrophages and inflammatory diseases by using PDK2 gene knockout mice, rather than using PDK2 inhibitors. In addition, the expression of PDK2 increased more than that of other PDK isoenzymes during osteoclast differentiation, and in ovariectomized PDK2-deficient mice, bone loss decreased and the number of osteoclasts reduced. Osteoclast differentiation is inhibited in PDK2-deficient bone marrow-derived monocyte/macrophage lineage cells, and inhibition of PDK2 prevents ovariectomization-induced bone loss (56). Currently, the roles of PDK2 in regulating the activation, proliferation, differentiation, and function of immune cells, including macrophages, dendritic cells, and T and B lymphocytes need further investigation.

### 5.3 Regulation of macrophage activation by PDK4

As a member of the PDK isoenzyme family, PDK4 regulates mitochondrial pyruvate metabolism (57–59) and is closely associated with the regulation of macrophage activation. HIF-1α is the key factor that regulates the cell response to hypoxia; it is activated under low oxygen conditions and can regulate various physiological activities, such as oxygen metabolism and glycolysis (60). High glucose upregulates the transcriptional activity and protein expression of HIF-1α (61). In atherosclerosis, anaerobic metabolism and pro-inflammatory activation of macrophages is dependent on HIF-1α (62, 63), and a change in HIF-1α protein levels directly affects the expressional level of PDK. Gene silencing of HIF-1α with siRNA decreases PDK4 expression; however, HIF-1α expression is unaffected by PDK4 (64). PDK4 expression is upregulated after HIF-1α directly binds to the promoter region of PDK4. Macrophages stimulated by advanced glycation end-products (AGEs) accelerate the pathological development of atherosclerosis. AGE-bovine serum albumin promotes M1 phenotype polarization by the HIF-1α/PDK4 pathway (65, 66). As a feedback regulator of pyruvate dehydrogenase, PDK4 plays a key function in glucose energy metabolism by participating in different signal transduction cascades (10, 21, 67, 68). Accumulating evidence shows that microRNAs (miRNAs) mediate macrophage activation in some diseases (69–72), and PDK4 plays a role in the regulation of macrophages by miRNAs.

As small and effective post-transcriptional regulators of correlated gene expression, miRNAs regulate the entire genetic network and are involved in multiple cellular processes. MiRNA dysregulation has been implicated in various human diseases (73, 74). MiR-33 affects cellular metabolism by regulating genes related to cholesterol transport, fatty acid metabolism, and insulin signaling (75–78). Cholesterol efflux by macrophages is controlled by ATP production in mitochondria, and the suppression of endogenous miR-33 promotes mitochondrial metabolism, resulting in cholesterol efflux from macrophages and the alleviation of atherosclerosis by targeting PDK4 and other genes (79). MiR-15a-5p plays crucial functions in multiple diseases including endometriosis, sepsis, and acute myocardial infarction (80, 81). Zhu et al. (82) established an animal model of sepsis by cecal ligation perforation. They confirmed the binding of miR-152-3p to PDK4, which resulted in the downregulation of PDK4 expression. MiR-152-3p suppressed LPS/IFN-γ-mediated mitochondrial autophagy of macrophages by directly targeting PDK4, alleviating inflammatory responses (83, 84).

The above studies show that PDK2 or PDK4 alone regulates macrophage activation by affecting cellular metabolism. Recently, the combined effects of PDK2 and PDK4 on macrophage activation and polarization have been investigated. Jha et al. carried out a series of experiments and their findings support that PDK2/PDK4 promotes M1 polarization after activation and that macrophage polarization influences the occurrence and development of chronic inflammatory hyperalgesia (85). Similarly, Min et al. reported that either PDK2/PDK4 gene combined deletion or inhibition of PDK2/PDK4 by small-molecule inhibitors suppresses the inflammatory response of macrophages after inflammatory stimulation (LPS and IFN-γ), downregulates the expression of M1 macrophage markers, affects bactericidal activity, adhesion, and migration of macrophages, alleviates insulin resistance induced by obesity, and improves adipose tissue inflammation (86). Macrophage infiltration induced by hyperglycemia and the release of pro-inflammatory cytokines in the dorsal root ganglia are considerably attenuated in PDK2/PDK4 gene-deficient mice (87). Because macrophages are the main cell population that infiltrate into inflammatory sites, PDK2-/PDK4-regulated macrophage polarization toward the pro-inflammatory M1 type provides a novel mechanism for the interaction between inflammation, chronic pain, and metabolism.

### 5.4 Regulation of the macrophage NLRP3 inflammasome by PDK

In macrophages, the NLRP3 inflammasome plays a crucial role in the defense against pathogenic invasions (88, 89) Abnormal activation of the NLRP3 inflammasome promotes cardiovascular dysfunction, type 2 diabetes, septicemia, novel coronavirus infection, and other inflammatory diseases (90–95). In septic mice, pharmacological inhibition of PDK blocks the activation of the NLRP3 inflammasome and subsequent caspase-1 activation, resulting in decreased production of IL-1β and other cytokines in macrophages. Furthermore, inhibition of PDK improves mitochondrial oxidative damage and dysfunction caused by the activation of the macrophage NLRP3 inflammasome (96). The data provide novel insights and directions for the application of PDK in the treatment of inflammatory diseases, including sepsis. However, in this study, different inhibitors were used to examine the regulation of metabolic pathways in the activation of the NLRP3 inflammasome; therefore, the interference of non-specific inhibitor effects cannot be excluded from the experiment. Since a pathway-specific inhibitor was used to block each targeted pathway and detect the effect on the NLRP3 inflammasome, there might be a combined effect of multiple pathways on PDK-mediated NLRP3 inflammasome activation.

Other studies suggested that double deletion of PDK2 and PDK4 led to the inhibition of metabolic pathways related to citrate and succinate accumulation (6, 97, 98) and that the intermediates of the mitochondrial TCA cycle, succinate (98, 99), itaconate (100–102), and fumarate (103), have different regulatory effects on the activation of the NLRP3 inflammasome. Owing to the complex regulatory mechanisms of PDK on the NLRP3 inflammasome, the influence of PDK on disease development via the regulation of the macrophage NLRP3 inflammasome needs to be further elucidated.

## 6 Discussion and conclusion

**Figure 2:**
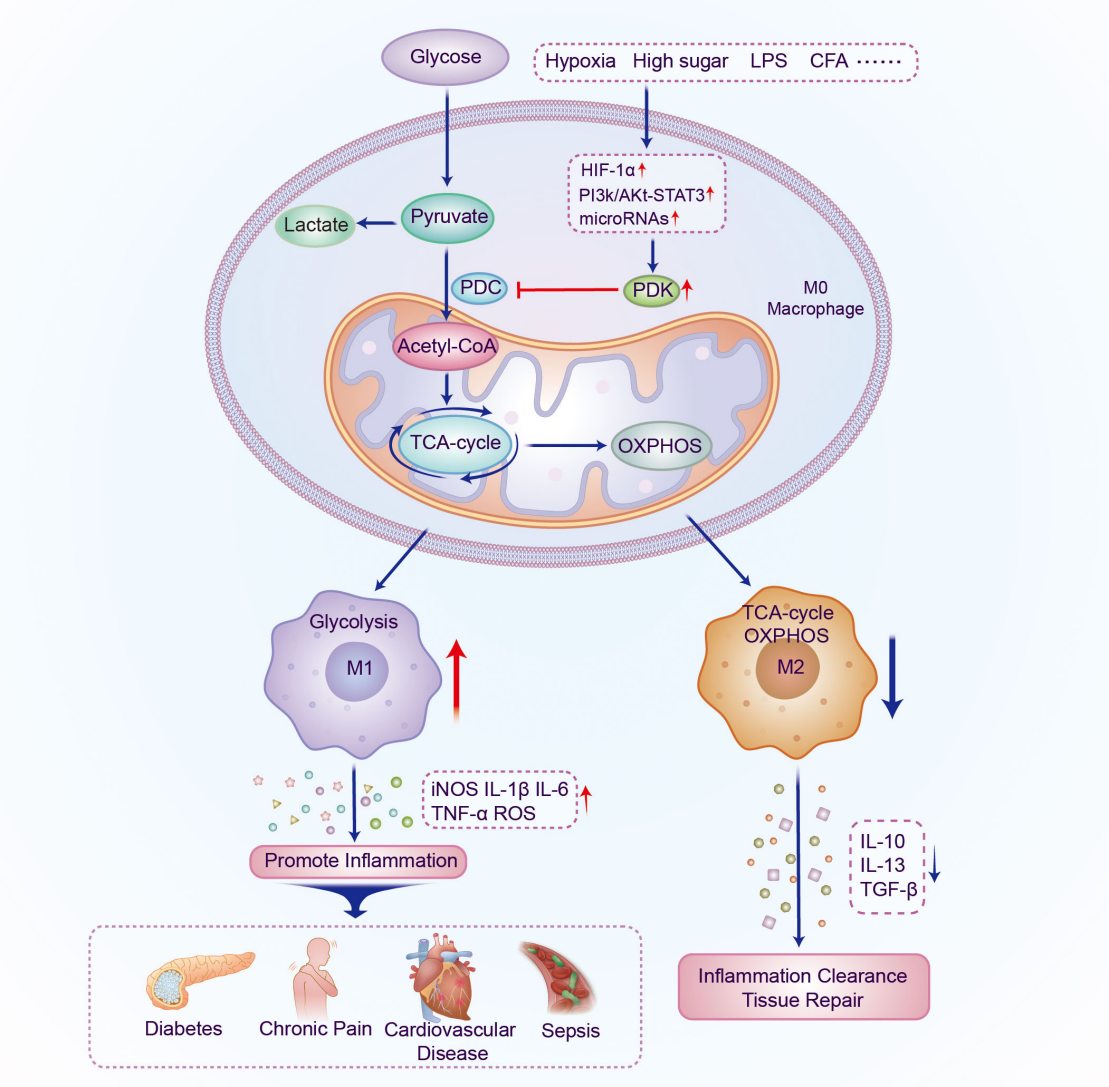
Schematic illustration of the mechanism by which PDK participates in regulating the polarization of macrophages. The expression of PDK is upregulated under the induction of different inflammatory factors; PDC activity and oxidative phosphorylation are inhibited and aerobic glycolysis increases. Macrophages polarize into the M1 phenotype and multiple pro-inflammatory genes are activated, thereby promoting the occurrence and development of metabolic and inflammatory diseases. PDK, pyruvate dehydrogenase kinase; PDC, pyruvate dehydrogenase complex; LPS, lipopolysaccharide; CFA, Complete Freund’s adjuvant; HIF-1α, hypoxia-inducible factor-1α; TCA, tricarboxylic acid; OXPHOS, oxidative phosphorylation; IL, interleukin; TGF, transforming growth factor.

In cells, metabolic reprogramming is essential for the activation, polarization, and function of macrophages. M1 macrophage energy metabolism is dominated by glycolysis of glucose to lactate, whereas M2 macrophages primarily rely on OXPHOS metabolic pathways. The two distinct macrophage phenotypes have different metabolic pathways and functions in inflammation-related diseases. This implies that targeting macrophage metabolism may be an effective way to combat inflammation-related diseases. PDKs regulate metabolic flexibility and contribute to energy homeostasis by negatively moderating PDC activity. In addition, PDK affects the polarization and immune response of macrophages by regulating the metabolic transformation from OXPHOS to aerobic glycolysis, and therefore plays a pivotal role in metabolic and inflammatory diseases (**Figure 2** and **Table 1**). Furthermore, small-molecule inhibitors of PDK have been identified, some of which are currently being investigated in animal models of metabolic and inflammation-related diseases. Although M1 and M2 macrophages have different ways of metabolizing energy, it remains unclear how these pathways contribute to the development of inflammatory diseases. Thus, further research is essential for demonstrating the relationship between PDK and macrophages and their potential as therapeutic targets for the treatment of metabolic and inflammatory diseases.

**Table 1:** PDKs regulate macrophage polarization in the disease state through various pathways.

PDK	Stimuli	Source	Mechanism	Response	Function	Role in disease	Ref.
PDK1	LPS, HKPA, PAM3CSK4	BMDMs, PEMs	Control glycolysis and glucose oxidation	IL-6, IL-12, IL-1β, iNOS	Promote M1 polarization and suppress M2 activation	Regulate macrophages activation	(41)
	mild hypoxia	RAW264.7 cell, PEMs	HIF-1α-PDK1 axis	Macrophage migration	Metabolic reprogramming affect macrophage migratory capacity	Suppress systemic inflammation	(46)
PDK2	LPS	RAW264.7 cell, THP-1 cell, BMDMs, PEMs	VSIG4-PI3K/Akt- STAT3 pathway	CD40, IL-6, TNF, IL-1β, L-12p40	Inhibit macrophage activation and M1 polarization	Ameliorate MHV-3- induced hepatitis	(54)
	LPS	BMDMs, BMDCs, macrophages	TLR4-MAPK pathway	CD86, CD40, pro- inflammatory factors	Promote proinflammatory response of macrophages and DCs	Accelerate endotoxin shock	(55)
	OVX	BMMs	RANKL-NFATc1 pathway	Osteoclast marker gene	Promote osteoblast differentiation	Improve ovariectomy-induced bone loss in mice.	(56)
PDK4	AGE-BSA	RAW264.7 cell	HIF-1α/PDK4 pathway	TNF-α, IL-6, IL-10, iNOS, Arg1	Induce M1 polarization	Involve in the development of diabetic atherosclerosis	(66)
	apoA1	THP-1 cell, BMDMs, PEMs	MiR-33 pathway	Cholesterol efflux	Regulatory macrophage cholesterol efflux	Protect from the development of atherosclerosis	(79)
	LPS	HPAEpiC, HSAECs	MiR-152-3p/PDK4, MiR-195-5p/PDK4	TNF-α, IL-6, IL-1β, cell viability, proliferation	Reduce the injury of pulmonary	Regulate sepsis- mediated ALI	(83, 84)
PDK2 and PDK4	CFA, LPS	PEMs, microglial cells	PDK-PDH-lactic acid axis	TNF-α, IL-1β, IL-6, Ym-1, Arg-1, IL-10, IRF4/8	Increase anti-inflammatory macrophages. promote glial activation	Promote chronic inflammatory insult	(65)
	LPS, HFD	BMDMs, PEMs	PDK-PDH-lactic acid axis	Emr1, Itgax, Tnf, Cd68, TNF-α, IL-6, IL-1β, iNOS	Increase proinflammatory effectors secretion	Promote adipose tissue inflammation and improve glucose tolerance in HFD-fed mice	(86)
	STZ	DRG, microglial cells	Glucose-PDK2/4- PDH-lactic acid axis	Macrophage infiltration, TNF-α, IL-1β, IL-6	Increase macrophage infiltration, satellite glial cells activation and proinflammatory cytokine release	Involve in the development of painful diabetic neuropathy	(87)
PDK	LPS, LPS+ATP, CLP	BMDMs, PEMs, PBMCs-derived macrophages	NLRP3 inflammasome	IL-1β, TNF, IL-6, caspase-1, metabolic analysis	Inhibit glucose flux and mitochondrial respiration	Promote acute inflammation	(96)

HKPA, heat-killed pseudomonas aeruginosa; BMDMs, bone marrow−derived macrophages; BMDCs, bone marrow−derived dendritic cells; OVX, ovariectomy, PEMs, peritoneal exudative macrophages; VSIG4, V-set immunoglobulin-domain-containing 4; BMMs, bone marrow–derived monocyte/macrophage lineage cells; AGE, advanced glycation end-products; BSA, bovine serum albumin; apoA1, apolipoprotein A-I; HPAEpiC, human pulmonary alveolar epithelial cells; HSAECs, human small airway epithelial cells; CFA, Freund’s adjuvant; HFD, a high-fat diet; STZ, streptozotocin; DRG, the dorsal root ganglion; PBMCs, peripheral blood mononuclear cells; CLP, cecal ligation and puncture.

## Author contributions

CYL: Writing – original draft. CBL: Visualization, Writing – original draft. JZ: Funding acquisition, Supervision, Writing – review & editing. YL: Investigation, Writing – original draft. BJ: Investigation, Writing – original draft. HX: Writing – review & editing. CXL: Funding acquisition, Writing – review & editing.
